# High-performance hybrid supercapacitor-immobilized Wells–Dawson polyoxometalates on activated carbon electrodes†

**DOI:** 10.1039/d3ra04478e

**Published:** 2023-09-06

**Authors:** Madhusree J E, Pranay R. Chandewar, Debaprasad Shee, Sib Sankar Mal

**Affiliations:** a Materials and Catalysis Laboratory, Department of Chemistry, National Institute of Technology Karnataka Surathkal 575025 India malss@nitk.edu.in; b Department of Chemical Engineering, Indian Institute of Technology Hyderabad Kandi Sangareddy 502284 Telangana India

## Abstract

The nanofabrication of electroactive hybrid materials for next-generation energy storage devices is becoming increasingly significant as supercapacitor (SC) technology develops rapidly. The present study utilizes activated carbon (AC) templates reinforced with Wells–Dawson polyoxotungstates (POMs) to produce nanohybrid electrodes for high-performance supercapacitors. This study analyzes Wells–Dawson polyoxotungstates (P_2_W_18_) for the first time integrated with AC, and its structural and electrochemical performances are discussed. First, the electrochemical performances of symmetric supercapacitors were characterized in an acidic aqueous electrolyte (0.5 M H_2_SO_4_). It was observed that a supercapacitor cell containing the 5 wt% AC-P_2_W_18_ hybrid symmetric displayed a noteworthy specific capacitance of 289 F g^−1^ and a remarkable energy density of 40 W h kg^−1^. Moreover, 5% AC-P_2_W_18_ symmetric supercapacitor cells showed 89% cyclic stability over 4000 cycles. Three LED lights were charged onto the electrode. The LEDs continued to illuminate continuously for red until 160 seconds, yellow until 20 seconds, and blue until 10 seconds after removing the electrode from the electrochemical workstation, demonstrating the device's power and energy density.

## Abstract 2

In the fabrication of a novel three-symmetric supercapacitor cell, three different wt% of Wells–Dawson polyoxometalate (5%, 10% & 15% P_2_W_18_) were impregnated on activated carbon (AC). In order to accomplish the real application as a device, the AC-P_2_W_18_ supercapacitor cell is lit up by a blue LED.

## Graphical abstract

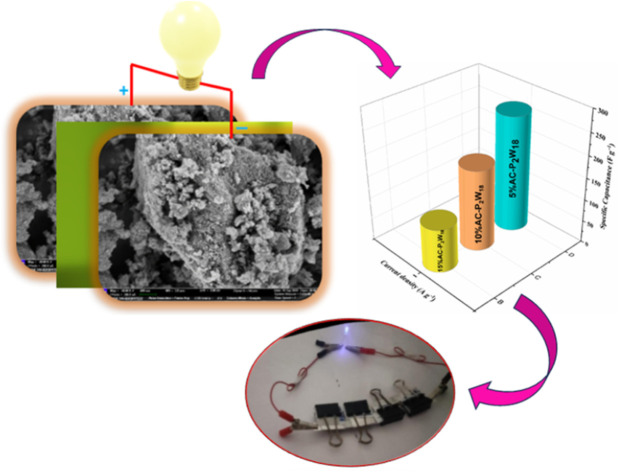

## 1. Introduction

The last few eras have seen rapid growth in several areas, such as industrialization and globalization. As a result, energy demands have increased exceptionally. In parallel, the human population has also grown, which accounts for much of the increase in energy consumption. Traditionally, fuels were used as energy sources, but fuel depletion led researchers to move toward electrochemical energy to replace them.^1,2^ The two primary energy power sources used for this were batteries and supercapacitors. Since supercapacitors can deliver higher power densities and longer cycle life than batteries with fast charge and discharge, they have gained significant attention.^3,4^ Supercapacitors were used in electronic devices, such as trains and buses, automobiles, cranes, and elevators.^5^ Double-layer capacitors (EDLC) and pseudocapacitors (PC) fall under the supercapacitor taxonomy. The electric doubler layer capacitor's energy storage and release mechanism rests entirely on the theory of charge separation at the electrode–electrolyte interface.^6^ As opposed to batteries, herein, there are no chemical oxidation-reduction reactions; thus, the nonfaradic nature of this physical charge transfer results in long cycling life.^7,8^ In EDLC, graphene-based,^9–11^ activated carbon,^12–14^ and carbon nanotube-based materials^15,16^ are typically used in the case of pseudocapacitors involved in the oxidation/reduction process. As a result, faradaic redox reactions have higher energy density.^17,18^ The materials used here include transition metal oxides,^19,20^ conducting polymers,^21,22^ metal sulfides,^23^ and polyoxometalates.^24,25^ The most usually used electrode material in commercial supercapacitors is activated carbon (AC) owing to its high surface area, low cost, high electrical conductivity, excellent corrosion resistance, high thermal stability, tuned pore structure, easy processability, and compatibility with other composite materials.^26^ However, because of its nonfaradic nature, the specific capacitance remains small. As a result, researchers began investigating new electrodes based on hybrid materials, the combination of an electric double layer, and a pseudocapacitor. It can boost the energy and power density while maintaining mechanical strength and the cell's long lifespan.

In this respect, inorganic metal–oxygen cluster polyoxometalates (POMs) have emerged as promising pseudocapacitive materials with diverse properties.^27^ The most unique property of POMs is that it can absorb and release electrons without changing its structural characteristics, which makes it an admirable supercapacitor electrode material. POMs comprise a multimetal oxide of early transition elements that can be altered in shape, size, and composition. It is nontoxic and nonvolatile, has a large molecular size, and relatively high molecular weight. Different types of POMs, such as Keggin,^28^ Dawson,^29^ Lindquist,^30^ Waugh,^31^ Silverton,^32^ and Anderson,^33^ and their multidirection applications are reported. POMs have been used in various applications, including sensors, catalysis, medicine, and energy storage. Among these, Keggin and Dawson types of POMs have been extensively investigated because of their thermal and redox stability and can engage reversible multielectron transfer reactions.^34^ This electron transfer metal-oxide cluster type suits electrochemical capacitors (EC). However, poor conductivity, low surface areas, and high solubility in an aqueous solvent prevent the direct application of POMs as an electrode material.^35^ To decrease the solubility, POMs are often deposited on a high surface area carbonaceous support to yield a nanocomposite for EC electrodes. Carbonaceous materials such as graphene,^36^ graphene oxide,^9^ single-walled carbon nanotubes (SWCNT),^37,38^ and multiwalled carbon nanotubes^39,40^ have been studied mainly as a support for the POM composite electrodes. Keggin POM clusters were mainly immobilized on the carbonaceous materials and investigated extensively in EC, resulting in reduced solubility and enhanced capacitance over soluble POMs. The following Table 1 summarizes the results of a comprehensive literature survey. As a result, different polyoxometalates doped with AC were described and used as supercapacitor electrodes. In 2012, Ruiz V. *et al.* successfully anchored molybdenum-containing Keggin POM [H_3_PMo_12_O_40_] (PMo_12_) onto the AC, and their electrochemical study was conducted in 1 M H_2_SO_4_, resulting in a specific capacitance value of 160 F g^−1^ at 2 A g^−1^ in a three-electrode system.^41^ In 2014, the same group developed an extensive method for impregnating [H_3_PW_12_O_40_] (PW_12_) on AC. A combination of double layer and redox activity was discovered to increase the specific capacitance by 254 F g^−1^ at 10 mV s^−1^.^42^ Hu C. *et al.* are interested in further exploring the same material PMo_12_-AC in an ionic liquid electrolyte (1 M [Bmim]HSO_4_), which attained a specific capacitance of 223 F g^−1^ at 1 mV s^−1^.^43^ In a follow-up study by Genovese *et al.*, activated carbon from pinecone biomass was synthesized, and PMo_12_ was deposited onto the pinecone-derived AC surface. This resulted in a specific capacitance of 361 F g^−1^ at 10 mV s^−1^.^44^

**Table 1:** An overview of the electrodes based on polyoxometalate-activated carbon

Electrode	Electrolyte	Current density/scan rate	Cell configuration	Specific capacitance	Ref.
AC-PMo_12_	1 M H_2_SO_4_	2 A g^−1^	3-Electrode	160 F g^−1^	41
AC-PW_12_	1 M H_2_SO_4_	10 mV s^−1^	3-Electrode	254 F g^−1^	42
AC-PMo_12_	1 M [Bmim]HSO_4_	1 mV s^−1^	3-Electrode	223 F g^−1^	43
Pinecone carbon-PMo_12_	1 M H_2_SO_4_	10 mV s^−1^	3-Electrode	361 F g^−1^	44
AC-VMo_11_	0.25 M H_2_SO_4_	0.2 A g^−1^	2-Electrode	430 F g^−1^	45
AC-MnV_11_	0.1 M H_2_SO_4_	0.4 A g^−1^	2-Electrode	479.73 F g^−1^	46
AC-NiV_14_	0.5 M H_2_SO_4_	0.2 A g^−1^	2-Electrode	365 F g^−1^	27
AC-P_2_Mo_18_	1 M H_2_SO_4_	6 A g^−1^	3-Electrode	275 F g^−1^	47
AC-P_2_W_18_	0.5 M H_2_SO_4_	0.2 A g^−1^	2-Electrode	288.48 F g^−1^	This work

Maity S. *et al.* fabricated a symmetric cell using vanadopolymolybdates at the AC surface and achieved a capacitance of 430 F g^−1^ at 0.2 A g^−1^ in 0.25 M H_2_SO_4_ in a 2-electrode system.^45^ Next, Vannathan A. A. *et al.* developed manganopolyvandate into an AC matrix, and the electrochemical performance was measured on a symmetric device in 0.1 M H_2_SO_4_ electrolyte. The specific capacitance was observed at 479.7 F g^−1^ at 0.4 A g^−1^.^46^ The research has so far focused on Keggin-based polyoxometalates ranging from homopolyanions to heteropolyanions. In addition, Maity S. *et al.* synthesized Lindqvist polyoxometalate (NiV_14_) doped on AC surfaces and performed an electrochemical study in 0.5 M H_2_SO_4_, which showed a capacitance of 365 F g^−1^ at 0.2 A g^−1^ in a symmetric cell.^27^

In this respect, Wells–Dawson-type POMs have been overlooked, which may be due to the larger size and higher charges than Keggin ions. In 2015, Mu A. *et al.* synthesized the first Dawson-based composite of octadecamolybdodiphosphate ((NH_4_)_6_[P_2_Mo_18_O_62_]·14.2H_2_O) doped on an activated carbon surface and investigated its electrochemical performance. It showed a specific capacitance of 275 F g^−1^ at a 6 A g^−1^ current density in a three-electrode system.^47^ Till today, there is no supercapacitor work reported using Wells–Dawson POM. Hence, it was interesting to investigate Well–Dawson-type POMs for supercapacitor applications. Herein, we explored activated carbon (AC)-supported K_6_[P_2_W_18_O_62_]. *x*H_2_O (P_2_W_18_)-based electrode for the electrochemical supercapacitor in a two-electrode system. In this paper, we doped P_2_W_18_ onto the AC matrix in different concentrations (5 wt%, 10 wt%, and 15 wt%) to examine the maximum amount of P_2_W_18_ on AC porous surface, which can enhance its capacitive nature. A symmetric SC cell composed of 5 wt% AC-P_2_W_18_, 10 wt% AC-P_2_W_18_, and 15 wt% AC-P_2_W_18_ was synthesized, and its electrochemical performance was evaluated using a two-electrode system in 0.5 M H_2_SO_4_ electrolyte. It has been observed that 5 wt% AC-P_2_W_18_ exhibited an excellent specific capacitance of 289 F g^−1^ at 0.2 A g^−1^ with good energy and power density of 40 W h kg^−1^ and 1999 W kg^−1^.

## 2. Results and discussion

### 2.1 FTIR

**Fig. 1:**
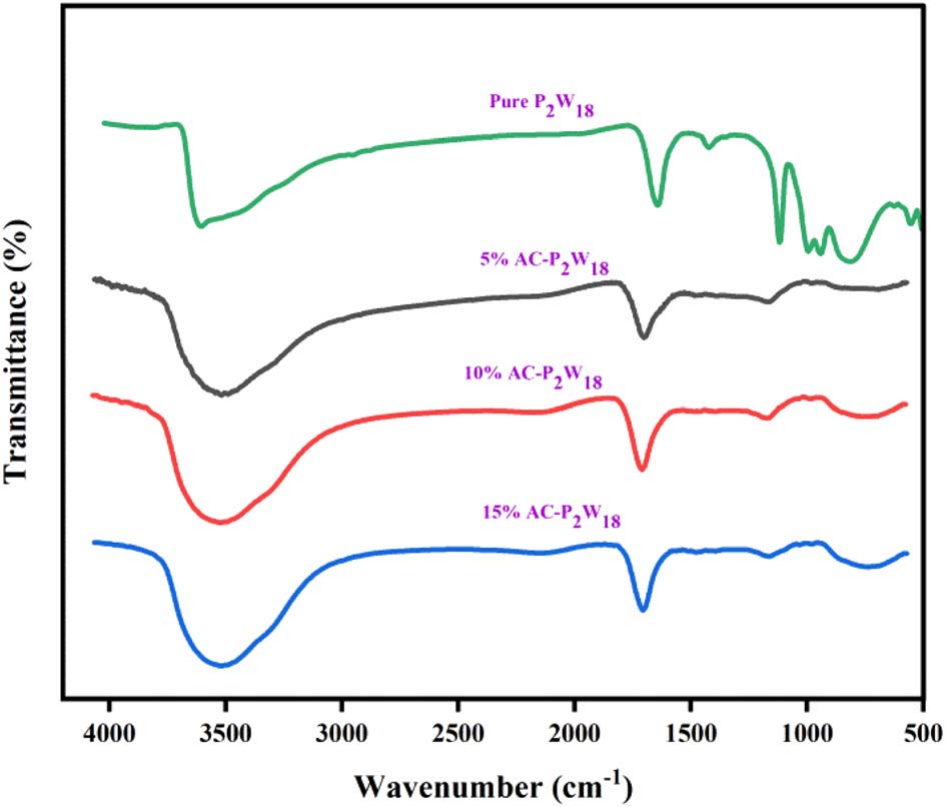
FTIR spectra of pure P_2_W_18,_ 5%, 10%, and 15% of AC-P_2_W_18_.

FTIR spectra were recorded on a Bruker 4000 spectrometer (USA) to determine the chemical structure of the materials. Fig. 1 represents the FTIR spectra of composites with different weight percentages and pure P_2_W_18_. The characteristic chemical bands in all the three composite materials and pure P_2_W_18_ correlated well with those reported in the literature.^48^ The bands at 1108, 824, 934, and 996 cm^−1^ of P_2_W_18_ are induced by vibrations of the P–O, W–Oe–W, W–Oc–W, and terminal W=O bonds (Table S1†). C=C stretching causes intensity bands at 1600 and 1700 cm^−1^, while the broad peak at 3500–3650 cm^−1^ is due to the –OH groups.^49^

### 2.2 XRD

**Fig. 2:**
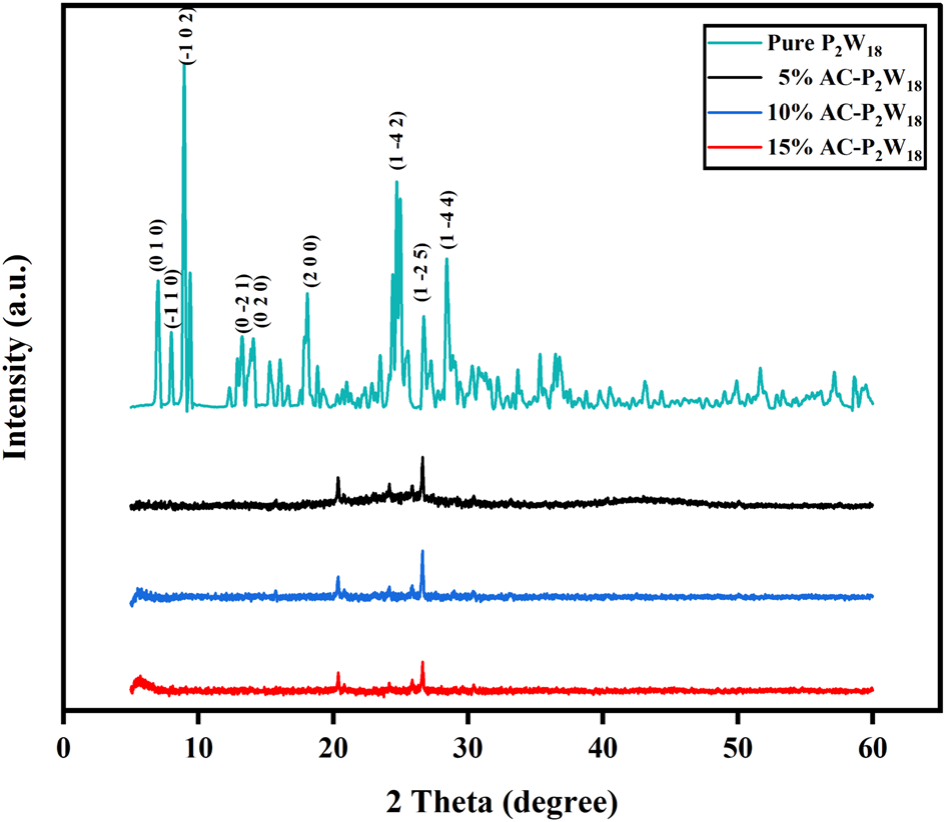
XRD patterns of pure P_2_W_18_, 5%, 10%, and 15% of AC-P_2_W_18_.

The powder XRD patterns of AC-P_2_W_18_ composites of different weight percentages are shown in Fig. 2. AC exhibits a broad pattern, indicating amorphous nature. The broad peaks observed at about 24° and 43.8° are due to graphitic carbon (0 0 2) and (1 0 0), respectively. The crystalline nature of P_2_W_18_ has been confirmed from the literature (Fig. 2)^48^ and JCPDS (card no: 01-073-6183). AC-P_2_W_18_ exhibits amorphous and crystalline characteristics as a result of P_2_W_18_ being incorporated in AC. The sharp peaks at about 20.35°, 24.13°, 25.85°, and 26.63° are due to P_2_W_18_. Pure P_2_W_18_ shows more intense peaks due to its well crystalline nature (Fig. 2). Conversely, in AC-P_2_W_18_, only a few intense peaks of P_2_W_18_ are visible because the carbon content is more in the nanohybrid than P_2_W_18_ and exhibits both amorphous and crystalline nature. The diffractions planes (0 0 2), (1 0 0), and (2 0 0) correspond to 2*θ* of 24, 43.8, and 20.35, respectively, for AC and P_2_W_18_.

### 2.3 XPS

**Fig. 3:**
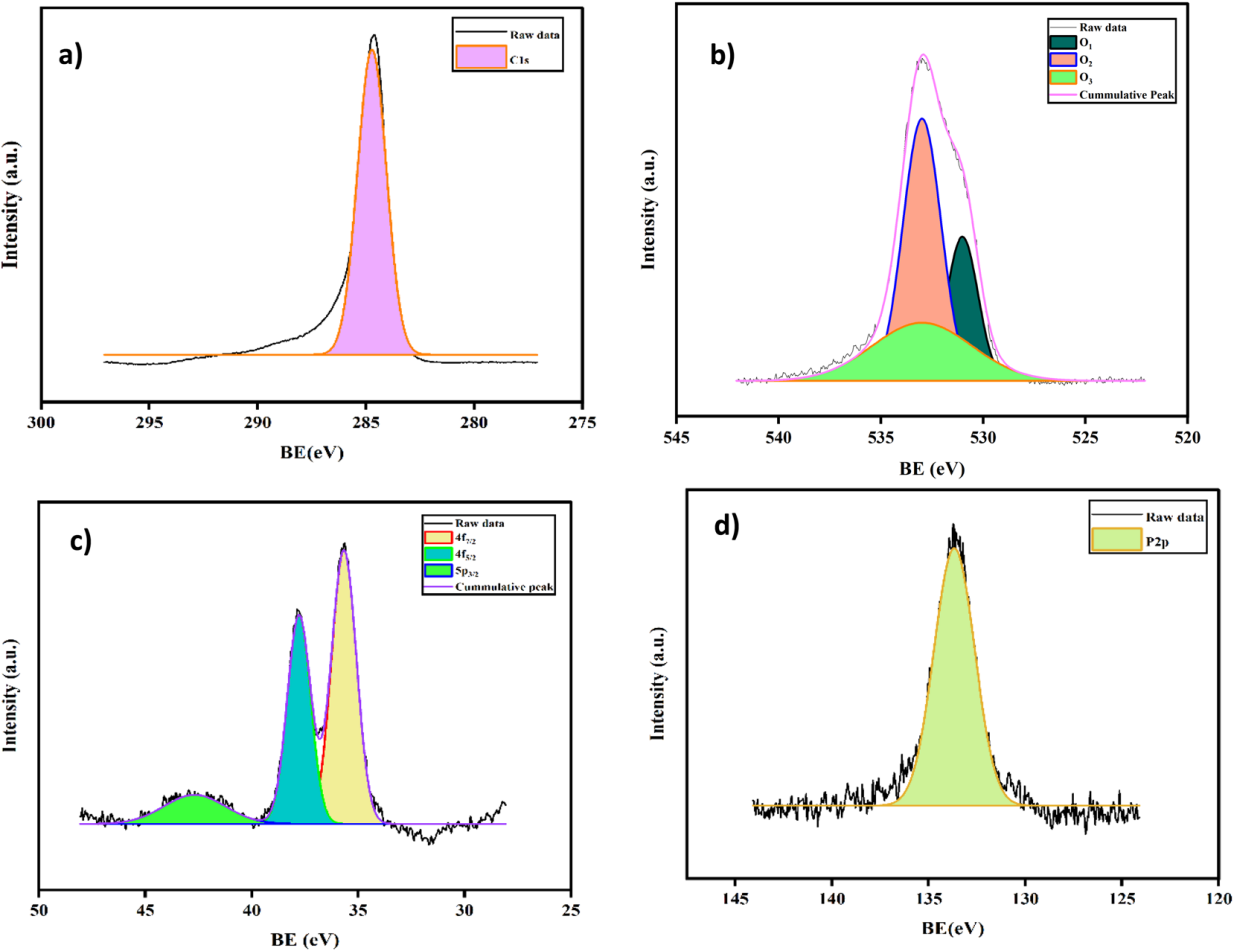
XPS spectra of AC-P_2_W_18_ (a) C 1s, (b) O 1s, (c) W 4f, and (d) P 2p.

X-ray photoelectron spectroscopy (XPS, Thermofisher scientific: Nexsa G2base) was used to probe the electronic states of the nanohybrids. Fig. 3 displays the high-resolution XPS spectra of 5% AC-P_2_W_18_. The survey spectrum shows the peaks of the elements found as C 1s, O 1s, W 4f, and P 2p for 5% AC-P_2_W_18_ in Fig. S1.† A single strong peak of C 1s measured at 285.3 eV, as shown in Fig. 3a, is consistent with the literature.^45^ A Gaussian function centered at 531.2 eV, 532.1 eV, and 533.6 eV due to W=O, O–W–O, and O–H, respectively, are satisfactorily fitted to the O 1s spectrum (Fig. 3b) of Well–Dawson polyoxometalate, which differs from the fit of Keggin polyoxometalate. A similar pattern applies to W, which is in the highest oxidation state of +6 (Fig. 3c), with peaks at 35.68, 37.5, and 42.71 eV, which correspond to 4f_7/2_, 4f_5/2_, and 5p_3/2_, respectively.^50^ Finally, a Wells–Dawson anion in P_2_W_18_ is attributed to the deconvoluted P 2p_3/2_ at 134.2 eV in Fig. 3d.

### 2.4 BET characterization

**Fig. 4:**
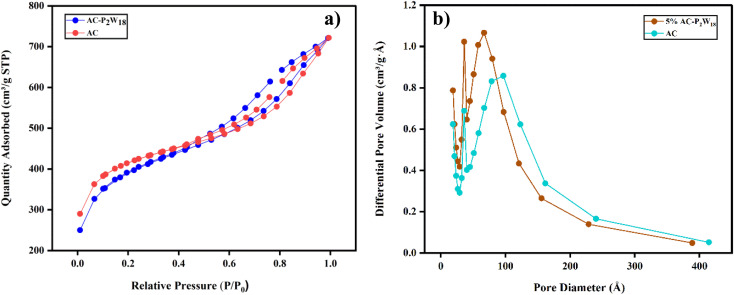
(a) N_2_ adsorption and desorption isotherms of AC and AC-P_2_W_18_, (b) pore size distribution of AC and AC-P_2_W_18_.

During electrochemical charge storage, electrode materials have an essential role in porosity and surface area. To characterize the AC and 5% AC-P_2_W_18_ nanohybrids, N_2_ adsorption/desorption isotherms data were collected using the Micromeritics physisorption analyzer (Model ASAP 2020, USA). The AC possesses the highest surface area of 1340 m^2^ g^−1^ with the highest pore volume of 0.37 cm^3^ g^−1^ (Table 2). A surface area of 1298 m^2^ g^−1^ and a pore volume of 0.25 cm^3^ g^−1^ was observed for 5% AC-P_2_W_18_, which clarifies that P_2_W_18_ is deposited on AC surfaces. The AC-P_2_W_18_ composite exhibited type-IV adsorption–desorption isotherms (Fig. 4a) with ill-defined hysteresis loops, suggesting the absence or less fraction of mesopores. The steep rise in volume adsorbed at low relative pressure indicates the presence of micropores. According to Fig. 4b, AC-P_2_W_18_ exhibits a pore size distribution peak at a higher pore diameter, indicating the presence of macropores. As a result, most micropores and some mesopores are covered by P_2_W_18_ on the AC surface.

**Table 2:** Surface area and porosity of the AC-P_2_W_18_ nanohybrid and AC

Sample	BET surface area (m^2^ g^−1^)	Micropore area (m^2^ g^−1^)	Micropore volume (cm^3^ g^−1^)	Mesopore width (Å)	Average nanoparticle size (Å)
AC	1339.8801	717.4	0.37	33.32	44.78
AC-P_2_W_18_	1297.9905	487	0.25	34.36	46.22

### 2.5 FESEM

**Fig. 5:**
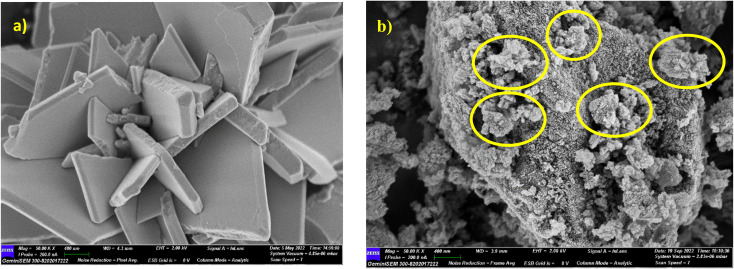
(a) FESEM image of pure P_2_W_18_, (b) 5% AC-P_2_W_18_.

The surface morphology of the nanocomposites was measured by FESEM (FESEM, Carl Zeiss Sigma, Germany). Fig. 5a and b show the FESEM images of pure P_2_W_18_ and 5% AC-P_2_W_18_ nanocomposites. It is clear from Fig. 5a that the pure P_2_W_18_ has a rock-like structure. The morphological study of pure AC has already been published in the literature by our group.^46^ An analysis of the surface morphology of 5% AC-P_2_W_18_ composite indicates that pure polyanions are inserted into the micropores of AC surfaces. Energy-dispersive spectroscopy (EDS) analysis was also carried out to identify the elements present in the composites. Fig. S2a† shows the confirmed elemental compositions of pure P_2_W_18_ of K, O, P, and W. The elemental compositions of the composites (K, P, O, W, and C) are also confirmed by EDS results (Fig. S2b†). In addition, HRTEM was evaluated to determine the microstructure of nanohybrids. As illustrated in Fig. S3a and b,† the well-decorated polyanions of P2W18 in the AC micropores can be seen in the micrograph of 5% AC-P2W18.

#### 2.5.1 Cyclic voltammetry

**Fig. 6:**
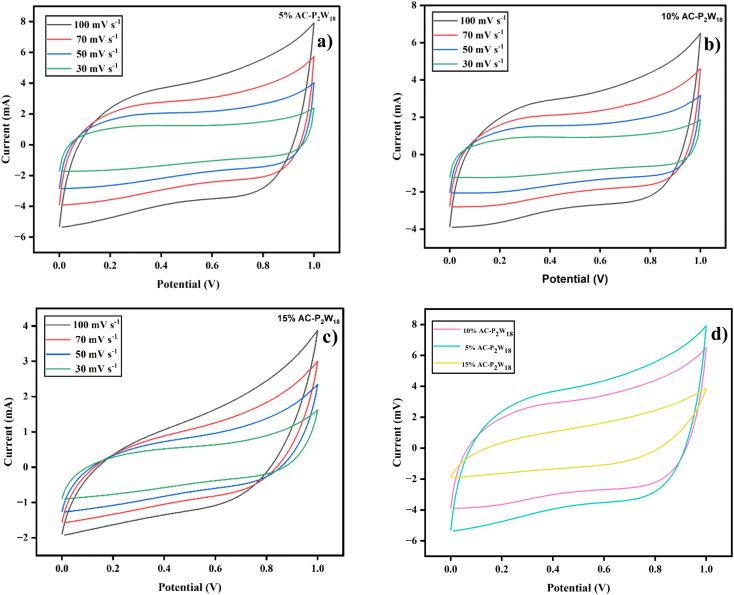
Cyclic voltammetry graphs (a) 5% AC-P_2_W_18_, (b) 10% AC-P_2_W_18_, (c) 15% AC-P_2_W_18,_ and (d) comparison graph of all three symmetric SC cells.

A two-electrode system of cyclic voltammetry (CV) method was utilized to explore the electrochemical properties of the power cell. An experiment using cyclic voltammetry (CV) measurements was done to study the electrode material's chemical kinetics, degradation process, and specific capacitance.^46^ The electrodes were tested in a cyclic (IVIUM Technologies BV Co., The Netherlands, Model: Vertex) setup using 5% AC-P_2_W_18_, 10% AC-P_2_W_18_, and 15% AC-P_2_W_18_ composite materials in the potential window of 0–1 V using 0.5 M electrolyte solution at various scan rates. A CV graph measuring 5% AC-P_2_W_18_, 10% AC-P_2_W_18_, and 15% AC-P_2_W_18_ has been presented in Fig. 6a–c at different scan rates of 30, 50, 70, and 100 mV s^−1^. CV analysis indicates that AC-P_2_W_18_ oxidation-reduction peaks of 5% and 10% indicate that is P_2_W_18_ present over AC surfaces. It is noticeable that 15% AC-P_2_W_18_ shows a deformed curve compared to the other two, 5% AC-P_2_W_18_ and 10% AC-P_2_W_18_. To better understand the pseudo material's exact oxidation-reduction behavior, the bare P_2_W_18_ electrode was also used to perform CV (Fig. S4†) in the same electrolyte at the same scan rate as the pseudomaterial. This indicates that the AC-P_2_W_18_ composite has an excellent capacitive response. An electrode's capacitance can be assessed using CV as can the shape of the cathodic and anodic peaks and the current density area (Fig. 6d). To evaluate the specific capacitance of nanohybrids, the CV plots were analyzed using eqn (1).1
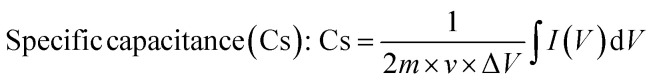
where *m*, *v*, and Δ*V* are the active material's mass, scan rate, and potential window, respectively. The specific capacitance increases when P_2_W_18_ is impregnated on AC surfaces (Tables S2–S4†).

#### 2.5.2 Galvanostatic charge and discharge

**Fig. 7:**
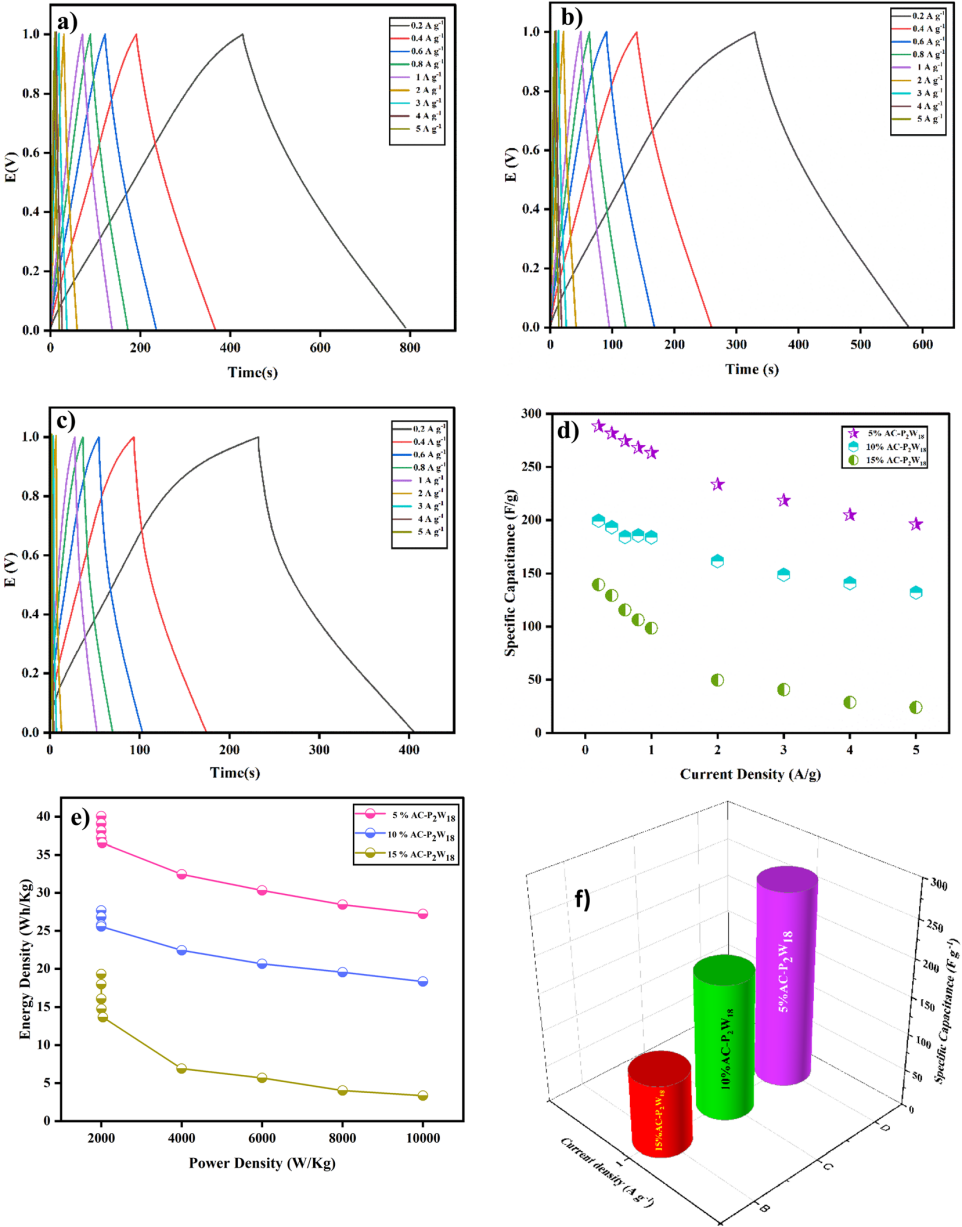
GCD graphs of (a) 5% AC-P_2_W_18_, (b) 10% AC-P_2_W_18,_ and (c) 15% AC-P_2_W_18_, (d) specific capacitance *vs.* current density for 5%, 10%, and 15% of AC-P_2_W_18_, (e) Ragone plot for all three nanohybrids and (f) comparison graph for all three symmetric SC cells.

Galvanostatic charge–discharge (GCD) at several current densities was examined to understand the electrochemical performance of the AC-P_2_W_18_ composite electrode materials under a defined potential window.^48^ In a galvanostatic charge and discharge study, three symmetric SC cells were examined with current densities in the range from 0.2 to 5 A g^−1^ in the potential window of 0–1 V. The charge–discharge outline was reshaped by immobilizing POMs onto the AC surface, which differs from the EDLC behavior. The improper linear GCD curve was observed across lower current densities.^46^ At 0.2 A g^−1^ current density (Fig. 7a), the 5% AC-P_2_W_18_ electrode exhibited a specific capacitance of 289 F g^−1^ with an energy density of 40 W h kg^−1^. The GCD graphs of 5% AC-P_2_W_18_ has showed the redox reaction while charging and discharging. To achieve higher energy density, the device's power must be compromised.^44^ In contrast to 10% and 15% of AC-P_2_W_18_, 5% AC-P_2_W_18_ consistently shows high power and energy densities (Fig. 7e) in the current density range from 0.2 to 5 A g^−1^. The specific capacitances of 5% AC-P_2_W_18_ with its energy and power densities are tabulated in Table S5†. Using eqn (2)–(4), we calculated the composite material's specific capacitance, energy, and power density. Meanwhile, the GCD response of 10% AC-P_2_W_18_ and 15% AC-P_2_W_18_ were recorded, which shows the specific capacitance value of 199 F g^−1^ and 139 F g^−1^ at 0.2 A g^−1^ current density with specific power and energy density values of 28 W h kg^−1^, 1999 W kg^−1^ and 19W h kg^−1^, 1999 W kg^−1^, respectively. The GCD graph response of 10% AC-P_2_W_18_ and 15% AC-P_2_W_18_ are displayed in Fig. 7b and c. The specific capacitance with their energy and power densities of 10% AC-P_2_W_18_ and 15% AC-P_2_W_18_ are tabulated in Tables S6 and S7†. Based on the GCD results on three different symmetric SC cells with the same current density, it is concluded that the 5% AC-P_2_W_18_-based electrode had a longer discharge time than the other two electrodes containing 10% AC-P_2_W_18_ and 15% AC-P_2_W_18_, resulting in a higher capacitance value for the former electrode. As a result, surface charge diffusion will be increased, leading to greater capacitance and energy density. In Fig. 7d, specific capacitance *versus* current and power *versus* energy density are plotted (Fig. 7e). It is observed that 5% AC-P_2_W_18_ gives high specific capacitance, high energy, and power densities compared to the other two, which explains that 5% AC-P_2_W_18_ is physisorbed more onto the AC surface. Fig. 7f illustrates that the 5% AC-P_2_W_18_ shows 18% higher capacitance than 10% AC-P_2_W_18_ and 45.5% higher than 15% AC-P_2_W_18_.2
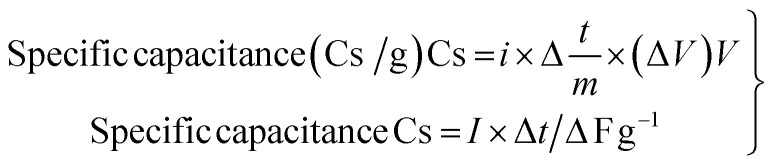

3 Energy density (E.D) *E* = *i*∫*V*d*t* W h kg^−1^

4
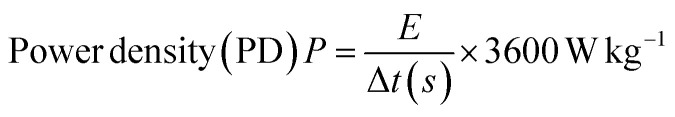


#### 2.5.3 Electrochemical impedance spectroscopy

**Fig. 8:**
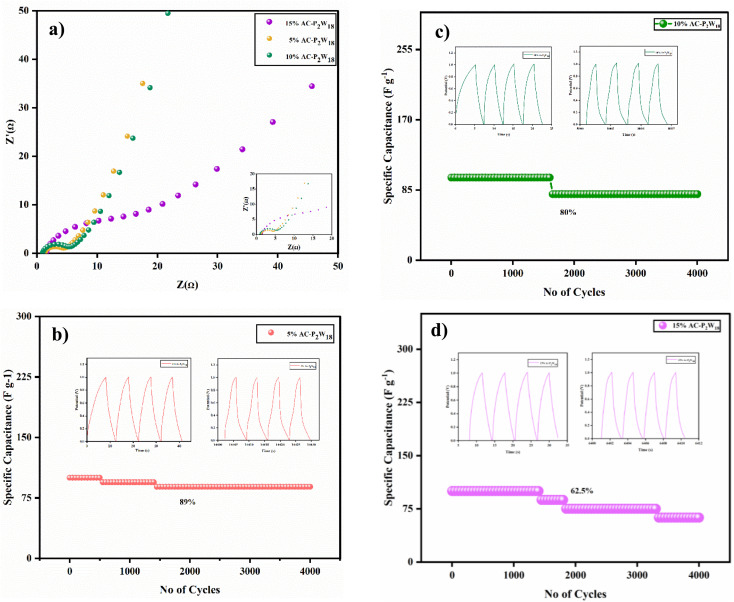
(a) Nyquist plot and cyclic stability of (b) 5% AC-P_2_W_18_, (c) 10% AC-P_2_W_18_, and (d) 15% AC-P_2_W_18_.

Power-cell impedance was measured using a low-amplitude dc potential with electrochemical impedance spectroscopy (EIS). All three different concentrations of P_2_W_18_ were deposited on AC and AC-P_2_W_18_ nanocomposites, and electrochemical impedance spectroscopy measurements were carried out using a dc potential of 0.01 V in the frequency range from 1 Hz to 100 kHz. Nyquist plots can be used to assess the internal resistance of the composites as well as their charge transfer kinetics and ion diffusion processes.^51^ The impedance spectroscopy results for the high-frequency region show the electrodes to be arranged in a semicircular arc. Low-frequency measurements of electrode–electrolyte impedance provide a visual representation of electron transfer kinetics of redox reactions due to limited mass transport.^52,53^ A partial semicircle was observed when the frequency increased, indicating the charge transfer resistance. As shown in Table S8,† all three different concentrations of AC-P_2_W_18_ nanohybrids exhibit equivalent series and charge transfer resistance. In the higher frequency regime, the RCT value is calculated from the diameter of the semicircle in the Nyquist plot (Fig. 8a). The *R*_CT_ value of 5% AC-P_2_W_18_ is lower at 3.35 (Fig. 8a) [Table S8†] than the other two, indicating that the smaller the electrode diameter, the greater the charge stored.^44^ Consequently, the 5% AC-P_2_W_18_ electrode has the highest conductivity and kinetics of charging compared to the other two electrodes.

#### 2.5.4 Cycle stability

**Fig. 9:**
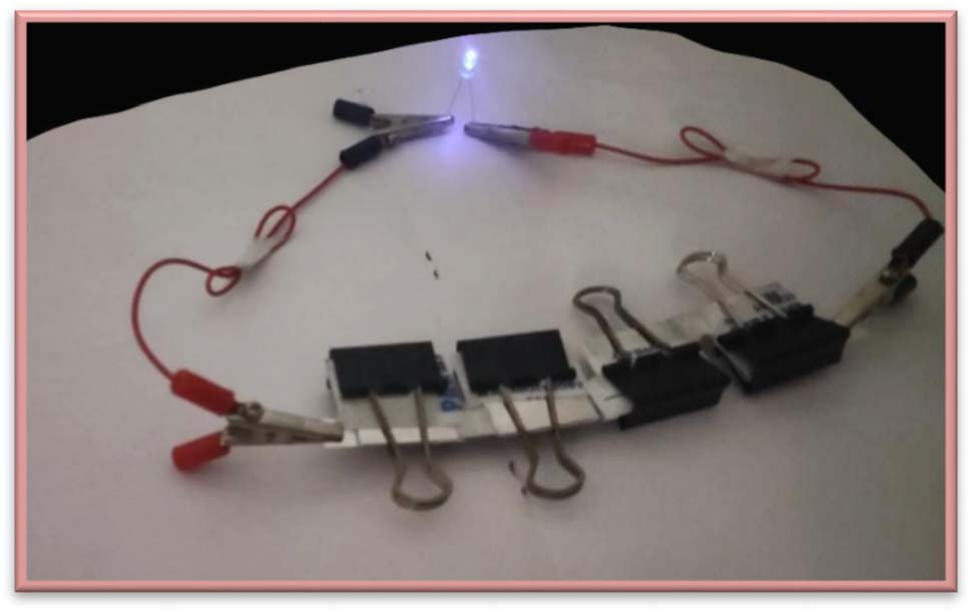
LED images of blue light using 5% AC-P_2_W_18_ as the electrode material.

To determine a supercapacitor device's application, it is essential to consider the cell's stability. Cycle stability has been tested on three symmetric electrodes composed of 5% AC-P_2_W_18_, 10% AC-P_2_W_18_, and 15% AC-P_2_W_18_ (Fig. 8b–d). As a result, in 4000 cycles at 7 A g^−1^ for a symmetrical electrochemical system with 5% AC-P_2_W_18_ electrode material (Fig. 8b), the electrochemical capacitors exhibited outstanding cycle stability of 89%, demonstrating that subsequent cycles do not affect long-term electrochemical capacitors but are similar to their initial cycle in terms of the cycle stability. The composite's first and last four cycles of cycle stability are exhibited in Fig. S5.† Based on the fabrication method described above, 5% AC-P_2_W_18_ was coated on four pairs of carbon clothes of 4 cm × 4 cm dimension (149 mg of active electrode material coated) were connected in a series. In the potential window of 0–3 V (Fig. 9, S6a and b†), the electrode was charged with three LEDs (red, yellow, and blue) at a high current density of about 20 A g^−1^ in an electrochemical workstation and lit up. After disconnecting the electrochemical workstation, the LED kept glowing continuously for the red one until 160 s, for yellow until 20 s, and for blue until 10 s after removing it (Video S1a–c†), proving the device's high energy and power density.

## 3. Materials and methodology

Activated carbon, *N*-methyl pyrrolidone (NMP), and sodium tungstate (Na_2_WO_4_) were purchased from Sigma-Aldrich. Orthophosphoric (85%), NH_4_Cl, KCl, distilled water, HPLC grade water, and methanol were all obtained from Loba Chemie. PVDF was purchased from Alfa Aesar. In this study, all analytical grade reagents were used without further purification.

### 3.1 Synthesis of the AC-P_2_W_18_ composite

The Wells-Dawson-type Polyoxometalate potassium octadecatungstate diphosphate K_6_[P_2_W_18_O_64_]·*x*H_2_O was synthesized according to the published procedure.^54^ To achieve different concentrations of P_2_W_18_ on AC, the amount of P_2_W_18_ was varied. AC-P_2_W_18_ was prepared using the procedure outlined below by varying the amounts of P_2_W_18_ of 5 wt%, 10 wt%, and 15 wt%. 1 g AC in 0.010 L methanol was dispersed in a round bottom flask. The methanol solution was agitated for 10 minutes with a magnetic stirrer to ensure even distribution. To prepare the P_2_W_18_ solution, P_2_W_18_ was dissolved in a small quantity of water (<5 mL) and then added dropwise into the AC-methanol solution. The solution form was agitated for approximately 24 hours at room temperature. After drying under reduced pressure, the solution was rinsed with enough quantity of aqueous solution to remove the excess P_2_W_18_. The black residue was then collected and finally allowed to dry.

### 3.2 Electrode preparation and cell fabrication

Electrochemical performances were measured by taking 90% of the active material (composites such as 5 wt% AC-P_2_W_18_, 10 wt% AC-P_2_W_18_, and 15 wt% AC-P_2_W_18_) mixed with 10% PVDF (as a binder). The mixture was taken in a mortar and pestle to blend the materials after it was combined with *N*-methyl-2-pyrrolidone (NMP, solvent) to make a uniform slurry. Next, a uniform layer of paste was coated on a flexible carbon cloth (each measuring 1 cm × 1 cm). The carbon cloth was weighed before and after modifications, which allowed us to determine the actual mass of the electrodes. We calculated only the active substance's mass (equivalent to 0.9 mg). 0.5 M H_2_SO_4_ served as a proton-conducting electrolytes for SC cells, and Whatman filter paper was used as a separator, which was soaked in the electrolyte. Individually symmetric arrangements were constructed to conduct electrochemical studies. As part of the symmetric SC fabrication process, an electrolyte solution-soaked filter paper separated an equivalent number of identical components from the carbon cloth.

## 4. Conclusion

The novel electrode materials were produced *in situ* using simple adsorption methods. In this study, we developed a Dawson polyoxometalate (P_2_W_18_) impregnated on AC surface hybrid electrode material for supercapacitor applications. Furthermore, symmetric cells such as 5% AC-P_2_W_18_, 10% AC-P_2_W_18_, and 15% AC-P_2_W_18_ nanostructures were electrochemically tested in a 0.5 M H_2_SO_4_ electrolyte solution using the two-electrode configuration. The 5% AC-P_2_W_18_ symmetric cell shows an upgraded specific capacitance value of 289 F g^−1^ with a high energy density of 40 W h kg^−1^. 5% AC-P_2_W_18_ shows superior electrochemical faradaic charge storage performance compared to other symmetric cells, which indicates that P_2_W_18_ is firmly incorporated onto the AC surface. To determine a supercapacitor's application, the 5% AC-P_2_W_18_ electrochemical capacitors achieved cycle stability of 89% over 4000 cycles.

## Author contributions

Madhusree J. E.: visualization, investigation, writing – original draft, software. Pranay R. Chandewar: investigation, data curation. Debaprasad Shee: writing – review and editing. Sib Sankar Mal: conceptualization, methodology, writing – review and editing, supervision.

## Conflicts of interest

The authors declare that they have no known competing financial interests or personal relationships that could have appeared to influence the work reported in this paper.

## Supplementary Material

RA-013-D3RA04478E-s001
