# Supplementary material for: High-performance hybrid supercapacitor-immobilized Wells–Dawson polyoxometalates on activated carbon electrodes
Source: RSC Adv. 2023 Sep 6;13(38):26744–54. doi: 10.1039/d3ra04478e (PMC10481258; doi:10.1039/d3ra04478e)
Supplement: RA-013-D3RA04478E-s001 [file RA-013-D3RA04478E-s001.pdf]

Electronic Supplementary Material (ESI) for RSC Advances.
This journal is © The Royal Society of Chemistry 2023

# High-performance hybrid supercapacitor Immobilized Wells-Dawson Polyoxometalates on Activated Carbon electrodes

Madhusree J E [a], Pranay R. Chandewar [b], Debaprasad Shee [b], Sib Sankar Mal [a*]

[a] Materials and Catalysis Laboratory, Department of Chemistry, National Institute of Technology Karnataka, Surathkal 575025, India

[b] Department of Chemical Engineering, Indian Institute of Technology Hyderabad, Kandi, Sangareddy 502284, Telangana, India

*Correspondences should be addressed: malss@nitk.edu.in

**1. FTIR table:**

| Assignments | 5% AC-$P_2W_{18}$ | 10% AC-$P_2W_{18}$ | 15% AC-$P_2W_{18}$ | $P_2W_{18}$ |
|---|---|---|---|---|
| O-H | 3528 | 3535 | 3510 | 3613 |
| P-O | 1155 | 1161 | 1155 | 1108 |
| W-O-W (intra) | 817 | 822 | 817 | 824 |
| W-O-W (inter) | 947 | 953 | 947 | 934 |
| W=O (terminal) | 998 | 1004 | 998 | 996 |
| C=C | 1696 | 1707 | 1704 | - |

**Table S1:** FTIR Values of 5% AC-$P_2W_{18}$ ,10 % AC-$P_2W_{18}$ and 15 % AC-$P_2W_{18}$ and $P_2W_{18}$

**1.XPS Survey spectra:**

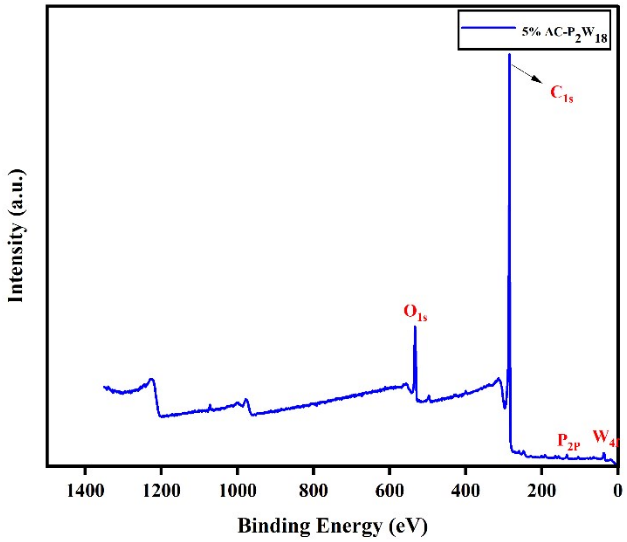


**Figure S1: XPS Survey of 5% AC-$P_2W_{18}$**

**3.EDS spectra:**

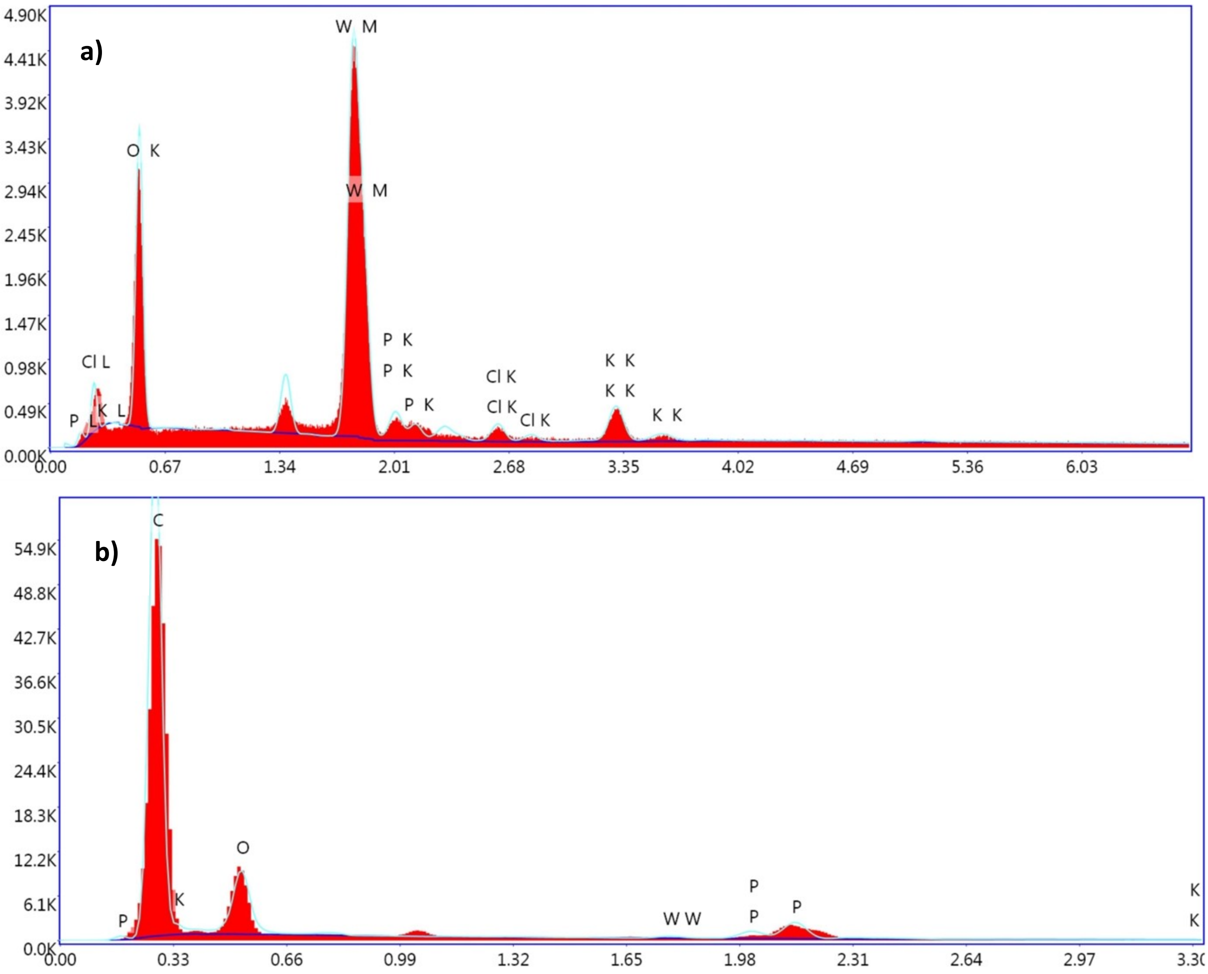


**Figure S2: EDS spectra of a) Pure $P_2W_{18}$ and b) 5% AC-$P_2W_{18}$**

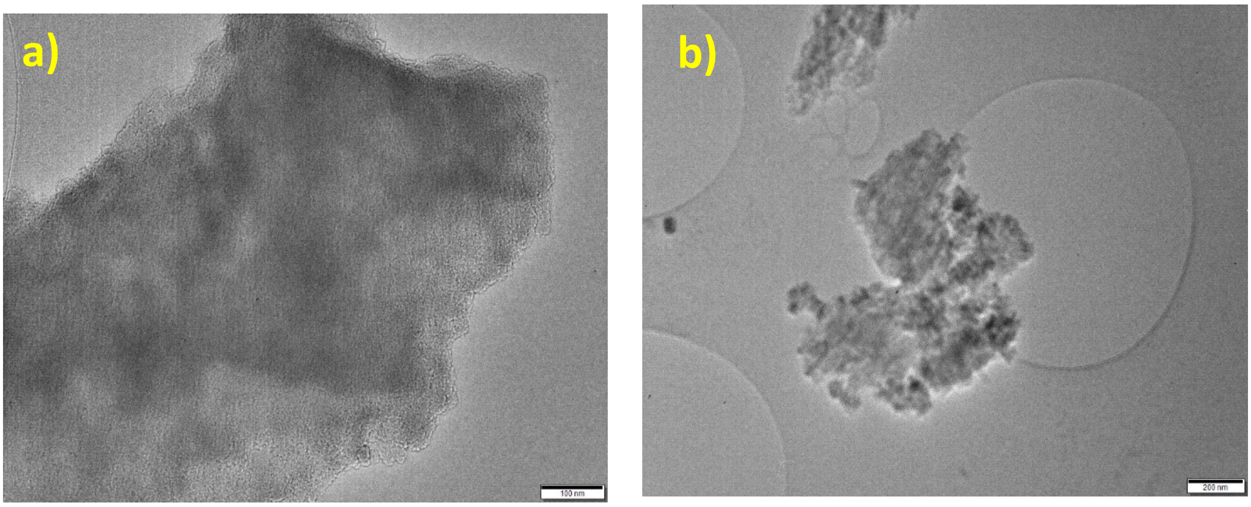


**Figure S3 : TEM images of a) 5%Ac-$P_2W_{18}$ magnification of 100nm b) ) 5%Ac-$P_2W_{18}$ magnification of 200nm**

**4.1. 5% AC-$P_2W_{18}$:**

| Scan rate(mV/s) | Potential(V) | Mass(mg) | Area (A) | Specific capacitance(F/g) |
|---|---|---|---|---|
| 100 | 1 | 0.9 | 6.1016 | 76.8916 |
| 70 | 1 | 0.9 | 4.6049 | 82.9179 |
| 50 | 1 | 0.9 | 3.4174 | 84.8088 |
| 30 | 1 | 0.9 | 2.1657 | 85.6948 |

**Table S2: Specific capacitance of 5% AC-$P_2W_{18}$ by cyclic voltammetry**

**4.2. 10% AC-$P_2W_{18}$:**

| Scan rate(mV/s) | Potential(V) | Mass(mg) | Area (A) | Specific capacitance(F/g) |
|---|---|---|---|---|
| 100 | 1 | 0.9 | 5.4558 | 60.6204 |
| 70 | 1 | 0.9 | 4.0114 | 63.6733 |
| 50 | 1 | 0.9 | 2.9291 | 65.0915 |
| 30 | 1 | 0.9 | 1.7436 | 64.5788 |

**Table S3: Specific capacitance of 10% AC-$P_2W_{18}$ by cyclic voltammetry**

**4.3. 15% AC-$P_2W_{18}$:**

| Scan rate(mV/s) | Potential(V) | Mass(mg) | Area (A) | Specific capacitance(F/g) |
|---|---|---|---|---|
| **100** | **1** | **0.9** | **2.10426** | **23.3806** |
| **70** | **1** | **0.9** | **1.71077** | **27.1539** |
| **50** | **1** | **0.9** | **1.36774** | **30.3933** |
| **30** | **1** | **0.9** | **0.95847** | **35.4988** |

**Table S4: Specific capacitance of 15% AC-$P_2W_{18}$ by cyclic voltammetry**

4.4: CV Graph of pure $P_2W_{18}$:

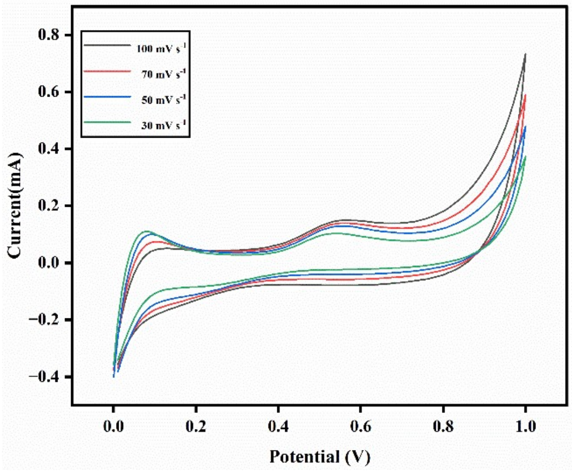


**Figure S4: CV graph of Pure $P_2W_{18}$**

**5. Galvanostatic Charge and Discharge Studies:**

**5.1. 5% AC-$P_2W_{18}$:**

| Current Density (A/g) | Specific capacitance (F/g) | Energy Density (Wh/kg) | Power Density(W/kg) |
|---|---|---|---|
| **0.2** | **288.48** | **40.066** | **1999** |
| **0.4** | **281.6** | **39.111** | **2000** |
| **0.6** | **274.56** | **38.133** | **2000** |
| **0.8** | **268.16** | **37.244** | **2000.95** |
| **1** | **263.2** | **36.555** | **2024.61** |
| **2** | **233.6** | **32.444** | **3999.94** |
| **3** | **218.4** | **30.333** | **5999.93** |
| **4** | **204.8** | **28.444** | **8000** |
| **5** | **196** | **27.222** | **9999.99** |

**Table S5: Specific capacitance, Energy density, and power density of 5% AC-$P_2W_{18}$**

**5.2. 10% AC-$P_2W_{18}$:**

| Current Density (A/g) | Specific capacitance (F/g) | Energy Density (Wh/kg) | Power Density(W/kg) |
|---|---|---|---|
| **0.2** | **199.36** | **27.688** | **1999** |
| **0.4** | **193.28** | **26.844** | **1999.66** |
| **0.6** | **184.32** | **25.600** | **2000** |
| **0.8** | **185.6** | **25.777** | **2000** |
| **1** | **184** | **25.555** | **2000** |
| **2** | **161.6** | **22.444** | **4000** |
| **3** | **148.8** | **20.666** | **6000** |
| **4** | **140.8** | **19.555** | **8000** |

| | | | |
|---|---|---|---|
| 5 | 132 | 18.333 | 10000 |

**Table S6: Specific capacitance, Energy density, and power density of 10% AC-$P_2W_{18}$**

**5.3. 15% AC-$P_2W_{18}$:**

| Current Density (A/g) | Specific capacitance (F/g) | Energy Density (Wh/kg) | Power Density(W/kg) |
|---|---|---|---|
| 0.2 | 139.09 | 19.311 | 1999 |
| 0.4 | 129.28 | 17.955 | 2000 |
| 0.6 | 115.68 | 16.066 | 2001 |
| 0.8 | 106.24 | 14.755 | 2003 |
| 1 | 98.4 | 13.666 | 2040 |
| 2 | 49.6 | 6.888 | 3999 |
| 3 | 40.8 | 5.666 | 5999 |
| 4 | 28.8 | 4.000 | 8000 |
| 5 | 24 | 3.333 | 9999 |

**Table S7: Specific capacitance, Energy density, and power density of 15% AC-$P_2W_{18}$**

**6. Cyclic Stability:**

**First Four Cycles**

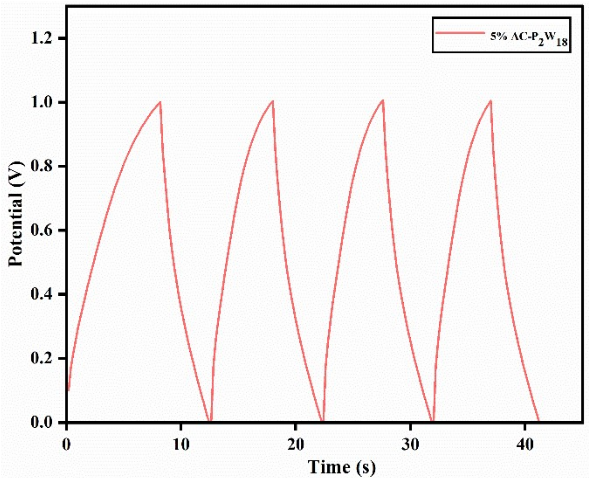


**Last Four Cycles**

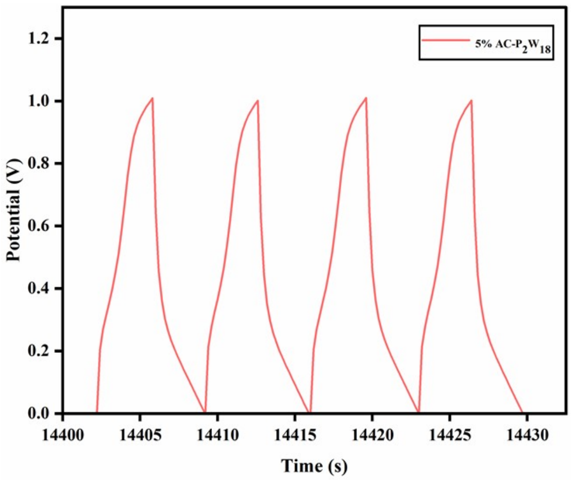

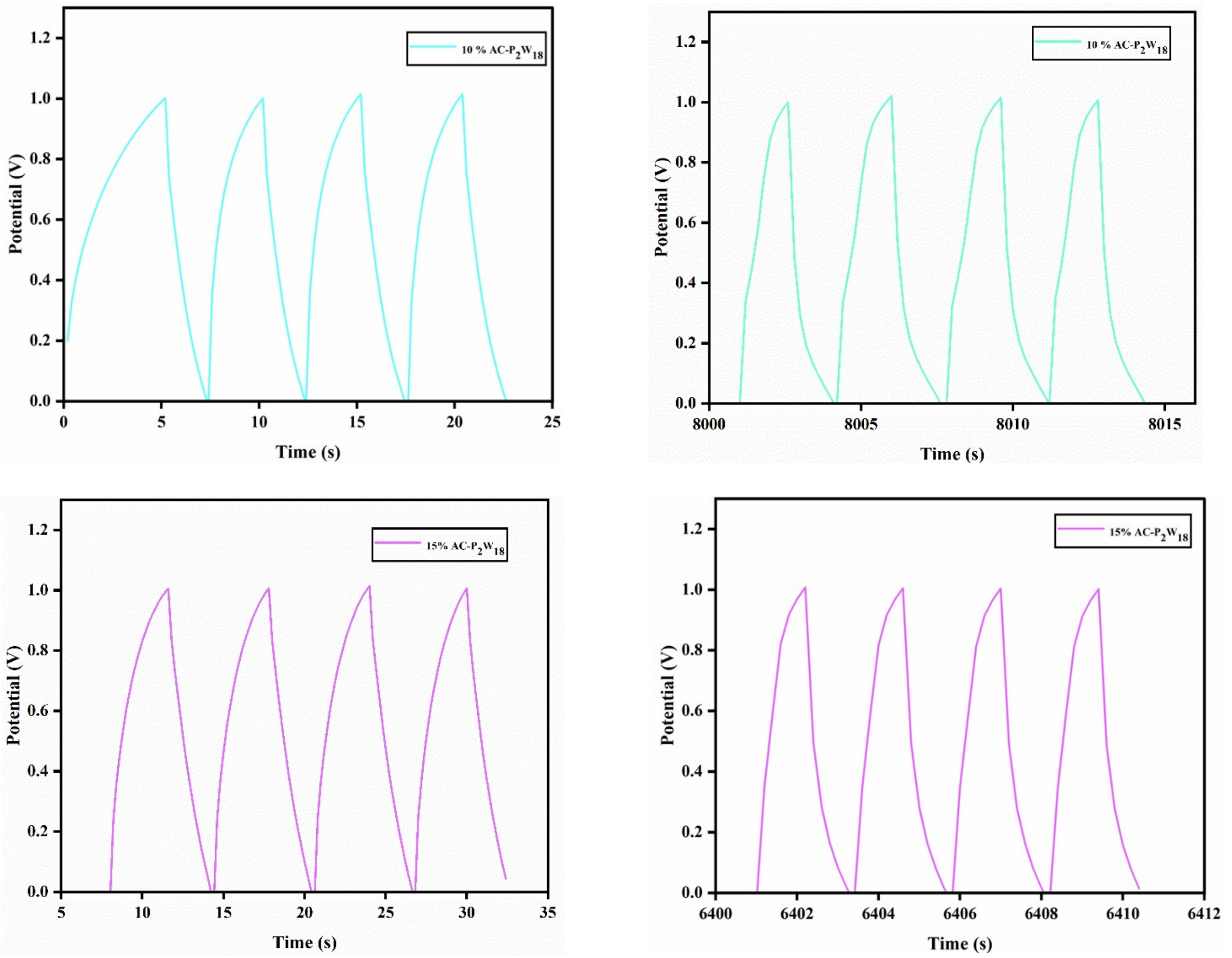


**Figure S5: First four cycles and Last four cycles of 5% AC-$P_2W_{18}$,10% AC-$P_2W_{18}$, and 15% AC-$P_2W_{18}$**

**7. Electro-Impedence Spectroscopy:**

| Materials | $R_S$ (Ω) | $R_P$ (Ω) | $R_{CT}$ (Ω) |
|---|---|---|---|
| **5% AC-$P_2W_{18}$** | 1.313 | 4.667 | 3.354 |
| **10% AC-$P_2W_{18}$** | 0.959 | 5.395 | 4.435 |
| **15% AC-$P_2W_{18}$** | 1.528 | 8.210 | 6.682 |

**Table S8: Equivalent circuit elements of all three 5% AC-$P_2W_{18}$, 10% AC-$P_2W_{18}$, and 15% AC-$P_2W_{18}$ plotted using Nyquist plots.**

**8. LED VIEDO:**

RED LED.mp4

Yelow LED.mp4

BLUE LED.mp4

**Video S1: a) RED LED, b) Yellow LED and C) Blue LED**

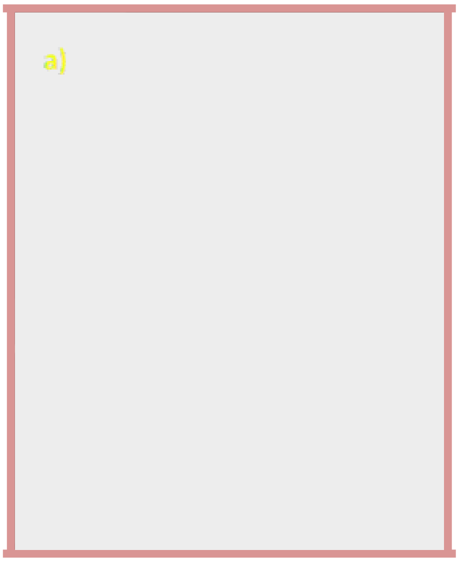


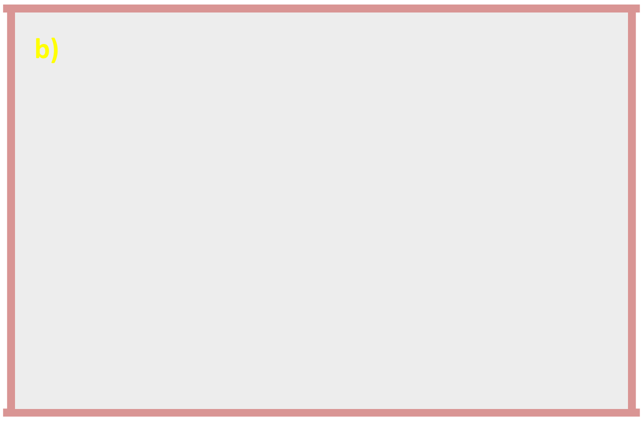

**Figure S6: LED images of a) blue b) Yellow light using the 5% Ac-$P_2W_{18}$ Electrode material**
